# Using machine learning to understand social isolation and loneliness in schizophrenia, bipolar disorder, and the community

**DOI:** 10.1038/s41537-024-00511-y

**Published:** 2024-10-05

**Authors:** Samuel J. Abplanalp, Michael F. Green, Jonathan K. Wynn, Naomi I. Eisenberger, William P. Horan, Junghee Lee, Amanda McCleery, David J. Miklowitz, L. Felice Reddy, Eric A. Reavis

**Affiliations:** 1 VA Greater Los Angeles Healthcare System, Los Angeles, CA USA; 2 VA Rehabilitation R&D Center on Enhancing Community Integration for Homeless Veterans, Los Angeles, CA USA; 3 Department of Psychiatry and Biobehavioral Sciences, Jane and Terry Semel Institute for Neuroscience and Human Behavior, UCLA, Los Angeles, CA USA; 4 Department of Psychology, UCLA, Los Angeles, CA USA; 5 Karuna Therapeutics, Boston, MA USA; 6 Department of Psychiatry and Behavioral Neurobiology, University of Alabama at Birmingham, Birmingham, AL USA; 7 Department of Psychological and Brain Sciences, University of Iowa, Iowa City, IA USA; 8 Department of Psychiatry, University of North Carolina, Chapel Hill, NC USA

**Keywords:** Human behaviour, Psychology

## Abstract

Social disconnection, including objective social isolation and subjective loneliness, is linked to substantial health risks. Yet, little is known about the predictors of social disconnection in individuals with mental illness. Here, we used machine learning to identify predictors of social isolation and loneliness in schizophrenia (*N* = 72), a psychiatric condition associated with social disconnection. For comparison, we also included two other groups: a psychiatric comparison sample of bipolar disorder (*N* = 48) and a community sample enriched for social isolation (*N* = 151). We fitted statistical models of social isolation and loneliness within and across groups. Each model included five candidate predictors: social avoidance motivation, depression, nonsocial cognition, social anhedonia, and social cognition. The results showed that social anhedonia explained unique variance in social isolation and loneliness in all samples, suggesting that it contributes to social isolation and loneliness broadly. However, nonsocial cognition explained unique variance in social isolation only within schizophrenia. Thus, social anhedonia could be a potential intervention target across populations, whereas nonsocial cognition may play a unique role in determining social disconnection in schizophrenia.

## Introduction

The health risks associated with social disconnection, which includes social isolation (i.e., the *objective* lack of social ties) and loneliness (i.e., the *subjective* feeling of having fewer social ties than desired), are substantial. Social isolation and loneliness are generally moderately associated; however, experiencing either can contribute to immune system dysregulation and an increased risk of early mortality^1,2^. While the exact prevalence rate of social disconnection in schizophrenia and other psychotic disorders is unclear, studies suggest that it could be as high as 80% —more than double that of the general population^3^. Knowledge of specific variables that relate to social isolation and loneliness is critical to mitigate their potential health consequences. Yet, little is known about the variables that are linked to social isolation and loneliness in mental illness and specifically schizophrenia.

To identify variables that are related to social isolation and loneliness in schizophrenia, it is critical to consider relevant variables from studies in serious mental illness (SMI) and the general population. Among individuals with SMI, research suggests links exist between isolation and nonsocial cognition, social cognition, and social anhedonia (i.e., lack of motivation for social engagement)^4–6^. In the general population, studies show that nonsocial cognition, social cognition, social avoidance (i.e., motivation to avoid negative social situations), depression, as well as isolation, predict loneliness^7–9^. Although relationships between those predictors and social isolation or loneliness have not been thoroughly examined in schizophrenia, social anhedonia could have particular relevance. Large-scale data-driven analyses in schizophrenia demonstrate that anhedonia is a central variable connecting multiple domains of social functioning^10–12^; however, the extent to which it explains unique variance in specific components of social functioning, such as social isolation and loneliness, is unclear.

In the current study, we used a novel recruitment strategy to help identify predictors of social isolation and loneliness across groups. We recruited a schizophrenia sample, a psychiatric comparison sample of bipolar disorder (BD), and a community sample (CS) enriched for social isolation. BD is an appropriate psychiatric comparison sample because, like schizophrenia, it is episodic and can have cycles of relapse and remission. In addition, those with BD typically are not as impaired on various clinical features, such as social cognition and social motivation, compared to people with schizophrenia^13^, but still show greater impairment than healthy controls^14^. Part of the CS was recruited using standard methods, but some community participants responded to online advertisements seeking individuals who self-identified as socially isolated. With this recruitment strategy, we obtained distributions of social isolation in the CS comparable to those in the schizophrenia and BD samples. Having similar distributions of social isolation enabled us to look at predictors in which the results were not confounded by group level differences in social isolation, which is common in studies of schizophrenia.

The wide range of variables that could impact social isolation and loneliness presents a challenge for interpretable data analysis. It is critical to account for interrelationships among variables and overall model complexity. Regression-based machine learning models utilizing a Least Absolute Shrinkage and Selection Operator (LASSO) account for such potential interrelationships among variables. Thus, with LASSO regression, we can test all possible combinations of variables and determine which combination results in the best-fitting model while avoiding overfitting^15^. Importantly, LASSO regression allows us to parsimoniously examine main effects and group interactions within the same model^16^.

Therefore, we used LASSO regression to examine the degree to which social cognition, nonsocial cognition, depression, social anhedonia, and social avoidance motivation were linked to social isolation and loneliness in schizophrenia, a psychiatric comparison sample (BD), and a CS enriched for social isolation. We evaluated the relationships among these variables that were present within samples and how the relationships differed between samples.

## Methods

### Participants

This study included 72 outpatients with schizophrenia, 48 with BD, and 151 community members. Participants were part of a larger study focused on the psychological components of social disconnection (RO1 MH110470 to MFG^17^). We recruited clinical samples from outpatient clinics at the Veterans Affairs Greater Los Angeles Healthcare System (GLA), the University of California, Los Angeles (UCLA), and outpatient facilities in the Greater Los Angeles area. We used the Structured Clinical Interview for DSM-5 (SCID-5^18^) for diagnoses. We corroborated diagnoses using medical records when available. All participants with schizophrenia and BD were clinically stable, with no hospitalizations within three months and no changes in psychoactive medication within four weeks. Both clinical samples were receiving psychoactive medications at the time of assessment.

To recruit community members high in social isolation, we posted ads asking: “Do you have few friends, little contact with family members, and typically do activities alone?” From these ads, we recruited 96 participants. We also ran online ads like those used in our previous studies in which healthy controls were unselected for level of social connection and tended to be socially connected^19^. In that ad, we did not mention social isolation. Fifty-five participants responded to those ads. Thus, the CS contained both isolated and non-isolated individuals and overall was enriched for isolation.

All participants in the CS provided psychiatric history through the SCID-5 and select sections of the SCID for Personality Disorders (SCID-PD^20^), which assessed for avoidant, paranoid, schizoid, schizotypal, and borderline characteristics. Participants were excluded if they met the criteria for a lifetime history of a psychotic disorder or bipolar disorder; however, personality disorder diagnoses were not exclusionary. Study procedures were approved by the Institutional Review Boards of GLA and UCLA, and all participants provided written informed consent.

Inclusion criteria for all samples were age 20–60 and understanding of English. Study candidates were excluded if they had (a) any clinically significant neurological disease, (b) a history of serious head injury (loss of consciousness > 1 h), (c) taken sedatives or benzodiazepines within 12 h of testing, (d) evidence of IQ < 70 or developmental disability based on the Wide-Range Achievement Test 3rd ed. reading subtest^21^, (e) substance use disorder at moderate level or greater in the past three months, or (f) current mood episode meeting criteria for depression, hypomania, or mania. Race data were collected by free response to provide description of the study sample.

### Clinical symptoms

Clinical symptom ratings were collected for all participants via semi-structured interviews. We used the Expanded Brief Psychiatric Rating Scale (BPRS^22^) to assess positive symptoms, the Hamilton Depression Scale (HAM-D^23^) to assess depressive symptoms, the Young Mania Rating Scale (YMRS^24^) to assess symptoms of mania, and the Clinical Assessment Interview for Negative Symptoms (CAINS^25^) to assess negative symptoms. We used the HAM-D total score as the measure of depression in all analyses.

### Social isolation and loneliness

To examine social isolation, we used a composite calculated from a previous paper by our group^17^. The composite is from three complementary scales: (1) Lubben Social Network Scale (12 item version^26^), (2) Social Disconnectedness Scale (last 4 items^27^), and (3) the Role Functioning Scale (social and family scores^28^). First, we standardized the three scales based on the values from a non-isolated community subgroup to anchor the scores to a typical healthy control sample. Second, we took the average of the standardized values to create the composite score. Third, we inversed scores so that larger values indicated greater social isolation. Further information on the calculation of the composite, including its reliability, is in ref. ^17^.

We measured loneliness using the UCLA Loneliness Scale (ULS Version 3^29^). The ULS is a 20-item self-report measure that assesses trait loneliness. Items are rated on a 1 to 4 scale, with higher scores indicating greater loneliness. We used a total score ranging from 20 to 80. The ULS had good internal consistency in all samples (ω = 0.90 in schizophrenia; ω = 0.86 in BD; ω = 0.92 in the CS).

### Social and nonsocial cognition

We used a composite of three measures of social cognition: mentalizing^30^, empathic accuracy^31^, and facial affect identification^32^. Mentalizing was assessed using the awareness of social inference test (TASIT) – Part 3. For this task, participants watched a series of videotaped vignettes that depict social interactions and answered four types of questions about the social interactions, including what a person: (a) believes or knows, (b) means, (c) intends, and (d) feels^30^. Sixteen vignettes are included, each with an untrue comment presented as either sarcasm or as a lie. A higher score indicates better mentalizing, with scores ranging from 0 to 64.

To measure empathic accuracy, we used a task from our prior studies^31^. Participants watched clips showing a person (a “target”) while they discussed a positive or negative autobiographical event. Participants used response keys to continuously rate how positive or negative they thought the target was feeling. The dependent measure was the mean correlation across clips between the participant’s ratings of the targets’ emotions and the targets’ ratings of their own emotions. A higher correlation indicated greater empathic accuracy.

We measured facial affect identification by having participants report the emotion in still photographs from a standardized stimulus set^32^. The test included photos of 8 different people displaying facial expressions of 6 emotions (afraid, angry, disgusted, happy, sad, and surprised) and a neutral expression. The dependent variable was percent accuracy, with higher scores indicating greater facial affect identification.

To create a composite measure of social cognition, we used principal component analysis. First, we standardized the variables using *z*-score transformation across the three samples. We then conducted a parallel analysis to determine the optimal number of components based on the covariance matrix structure. The parallel analysis indicated that the social cognitive variables optimally explained the proportion of variance in one component (variance explained = 0.556). The component loadings were high: TASIT = 0.818; empathic accuracy = 0.737; facial affect identification = 0.676. Higher scores indicated greater social cognitive ability.

Nonsocial cognition was assessed using the neurocognitive composite of the Measurement and Treatment Research to Improve Cognition in Schizophrenia Consensus Cognitive Battery (MCCB^33^). It measures 6 domains of cognitive functioning, including speed of processing, sustained attention, working memory, verbal learning and memory, visual learning and memory, and reasoning and problem solving. The composite was standardized to a T score based on national norms and corrected for age and gender.

### Social anhedonia and social avoidance motivation

We used a brief version of the Social Anhedonia Scale (SAS^34^) to measure social anhedonia. This version of the scale includes 24 true-false questions and was chosen based on a factor analysis that showed improved fit compared to the original scale^35^. The items measure trait social approach motivation. True responses are scored as 1, and false responses as 0. We used a total score ranging from 0 to 17, with higher scores indicating greater social anhedonia. Internal consistency of the SAS was acceptable in all samples (ω = 0.82 for schizophrenia; ω = 0.78 for BD; ω = 0.85 for the CS).

We used the Sensitivity to Rejection Scale^36^ to measure social avoidance motivation. This scale includes 24 items assessing 5 factors of trait social avoidance. Items are scored using a 9-point scale ranging from −4 to +4. We used a total score ranging from −96 to +96, with higher scores indicating greater social avoidance. The scale had acceptable internal consistency in all samples (ω = 0.76 for schizophrenia; ω = 0.83 for BD; ω = 0.81 for the CS)

### Data analysis

We performed all analyses using R (Version 4.2.0), with code available at OSF (https://osf.io/76ryh/). Regularized regression models using LASSO were our primary analyses. LASSO regression is a machine learning technique in which all possible combinations of numerous variables can be automatically estimated to obtain the overall best-fitting and parsimonious model. A key feature of LASSO regression is that the estimated best-fitting model does not retain independent variables that have limited value in relation to the dependent variable^15,16^. This approach can be particularly beneficial when multiple group interactions are included in the model. LASSO regression uses cross-validation to test the robustness of model results across subsets (i.e., “folds”) of participants to reduce model overfitting^15,16^.

All variables were standardized. First, we conducted within-group LASSO regression models (schizophrenia, BD, CS). For these models, we linked social isolation to loneliness, social cognition, nonsocial cognition, depression, social anhedonia, and social avoidance motivation across samples. In separate models, we linked loneliness from social isolation, social cognition, nonsocial cognition, depression, social anhedonia, and social avoidance motivation.

Next, we wanted to know how comparable the predictors were across samples. To check this, we examined main effects across groups and group-based interaction effects for all independent variables. The schizophrenia sample was used as the reference group because it was the main clinical group of focus. Hence, the interactions show whether the associations between the independent variables in BD and the CS and social isolation and loneliness differ relative to schizophrenia.

For the purposes of this study, all LASSO regression models were estimated using 5-fold cross-validation, in which the data were split into five equal parts, with the model built on 80% of the data and tested on the remaining 20%. We chose 5-fold cross-validation to balance model accuracy and complexity, consistent with prior research in schizophrenia^37^. The procedure involves testing a sequence of values that control the degree of regularization applied to the model coefficients (i.e., lambda values), thus preventing overfitting by penalizing (i.e., “shrinking”) large coefficients. The algorithm tests a sequence of 100 lambda values during the cross-validation process. These values determine the level of regularization applied to the model coefficients, starting from a maximum where all coefficients are zero to a minimum close to zero, enhancing the model’s fit to the data. The optimal lambda minimizes cross-validation error, providing a robust model. We conducted these analyses using the *glmnet* package^38^.

## Results

Table 1 contains descriptive statistics of sociodemographic and symptom data, and all variables used in the machine learning analyses. The groups had comparable levels of social isolation and did not statistically differ from each other [*F* (2, 268) = 1.59, *p* = 0.213, η^2^ = 0.012]; see Table 1 for the sample means. Twenty-seven CS participants met criteria for a personality disorder, including: 12 avoidant, 1 borderline, 4 paranoid, 5 schizoid, 1 schizotypal, 2 avoidant and schizoid, 1 paranoid and schizotypal, 1 schizoid and paranoid.

**Table 1:** Sociodemographic characteristics, clinical symptoms, and LASSO variables for schizophrenia, bipolar disorder, and community samples.

	Schizophrenia (*n* = 73)	Bipolar disorder (*n* = 48)	Community (*n* = 151)	*F* or *χ*^2^	*p*
Age	47.4 (11.3)	45.4 (10.8)	46.2 (10.3)	0.52	0.598
Education	12.9 (1.7)	14.5 (1.9)	14.8 (2.1)	24.09	<0.001
Race (*%*)				6.42	0.78
Asian	5.5	2.0	8.0		
Black	32.0	23.0	25.8		
Other	5.5	10.5	8.6		
Pacific Islander	0	2.0	1.0		
Native American	0	0	1.0		
White	57.0	62.5	55.6		
Sex (*%*)				0.32	0.848
Male	65.3	60.5	64.2		
Female	34.7	39.5	35.8		
Positive symptoms	2.4 (1.2)	1.2 (0.4)	1.1 (0.2)	97.51	<0.001
Expressive symptoms	1.0 (0.9)	0.5 (0.7)	0.3 (0.6)	25.89	<0.001
Motivation symptoms	1.7 (0.9)	1.3 (0.8)	1.1 (0.9)	10.90	<0.001
Maina symptoms	5.9 (5.5)	3.7 (4.8)	1.6 (2.2)	31.78	<0.001
Social Isolation^a^	0.3 (0.7)	0.2 (0.7)	0.3 (0.9)	1.59	0.213
Loneliness^a^	44.4 (9.6)	49.1 (10.8)	47.0 (11.7)	2.71	0.067
Depression^a^	7.3 (5.4)	8.2 (6.2)	4.1 (4.7)	16.52	<0.001
Social Cognition^a^	−0.9 (1.4)	0.2 (1.0)	0.4 (1.1)	28.01	<0.001
Nonsocial Cognition^a^	37.1 (14.4)	42.3 (11.5)	47.0 (11.5)	15.57	<0.001
Social Avoidance^a^	10.5 (13.8)	12.3 (19.9)	0.02 (22.5)	10.48	<0.001
Social Anhedonia^a^	6.1 (3.4)	6.8 (4.5)	7.1 (5.1)	1.10	0.344

*LASSO* Least Absolute Shrinkage and Selection Operator.

^a^Variables used in the LASSO regression analyses.

**Fig. 1:**
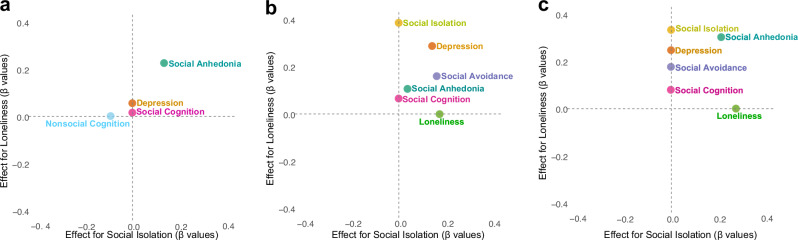
Within-group LASSO regression models of social isolation and loneliness in schizophrenia, bipolar disorder, and community samples. **a** Schizophrenia; (**b**) bipolar disorder; (**c**) community. LASSO, Least Absoluter Shrinkage and Selection Operator. Data points correspond to estimated β values for models predicting social isolation and loneliness.

The results for the within-group LASSO regression models are presented in Fig. 1. In schizophrenia (Panel a), the model of social isolation had an R^2^ of 0.14 and the model of loneliness had an R^2^ of 0.21. Social anhedonia (*β* = 0.14, % of runs = 100) and nonsocial cognition (*β* = −0.09, % of runs = 96.66) were the only variables that explained unique variance in social isolation in schizophrenia. For loneliness, social anhedonia (*β* = 0.22, % of runs = 100), social cognition (*β* = 0.01, % of runs = 85.00), and depression (*β* = 0.05, % of runs = 90.00) were non-zero predictors.

In BD (Panel b), the model of social isolation had an R^2^ of 0.48 and the model of loneliness had an R^2^ of 0.45. In contrast to schizophrenia, multiple variables explained unique variance in social isolation, including social anhedonia (*β* = 0.03, % of runs = 81.96), depression (*β* = 0.16, % of runs = 96.72), social avoidance (*β* = 0.16, % of runs = 95.08), and loneliness (*β* = 0.17, % of runs = 100). However, nonsocial cognition shrunk to 0. For loneliness, all variables except nonsocial cognition were non-zero predictors, including social anhedonia (*β* = 0.10, % of runs = 83.05), depression (*β* = 0.28, % of runs = 96.61), social avoidance (*β* = 0.19, % of runs = 86.44), social cognition (*β* = 0.08, % of runs = 67.80), and social isolation (*β* = 0.38, % of runs = 100).

In the CS (Panel c), the model of social isolation had an R^2^ of 0.40 and the model of loneliness had an R^2^ of 0.57. Like the schizophrenia and BD models, social anhedonia explained unique variance in both social isolation (*β* = 0.21, % of runs = 98.38) and loneliness (*β* = 0.31, % of runs = 100). Loneliness also explained unique variance in social isolation (*β* = 0.28% of runs = 100). In addition to social anhedonia, social cognition (*β* = 0.06, % of runs = 62.71), social avoidance (*β* = 0.18, % of runs = 88.13), depression (*β* = 0.25, % of runs = 93.22), and social isolation (*β* = 0.33, % of runs = 98.30) explained unique variance in loneliness. Nonsocial cognition was shrunk to 0.

**Fig. 2:**
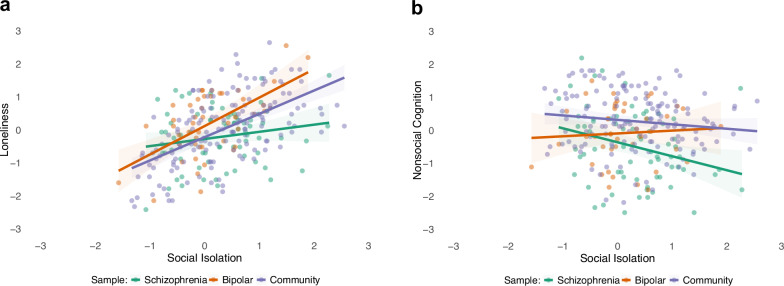
Scatterplots of social isolation and loneliness and social isolation and nonsocial cognition in schizophrenia, bipolar disorder, and a community samples. **a** Scatterplot of social isolation and loneliness; (**b**) scatterplot of social isolation and nonsocial cognition.

Next, we conducted the across-group analyses. For social isolation (Table 2), social anhedonia, loneliness, and nonsocial cognition showed non-zero main effects. There were non-zero interactions between social isolation and loneliness, such that the effect between these variables was stronger in BD and CS, relative to schizophrenia. There was also an interaction between social isolation and nonsocial cognition, such that the effect was weaker in BD relative to schizophrenia. For illustrative purposes, we present simple correlations corresponding to these interactions in bivariate scatterplots (Fig. 2). Panel a shows that social isolation and loneliness were strongly correlated in BD (*r* = 0.59, *p* < 0.001) and the CS (*r* = 0.58, *p* < 0.001) but not schizophrenia (*r* = 0.18, *p* = 0.124). Panel b shows that social isolation and nonsocial cognition were significantly correlated in schizophrenia (*r* = −0.29, *p* = 0.001) but not BD (*r* = 0.06, *p* = 0.679) or the CS (*r* = −0.13, *p* = 0.117).

**Table 2:** Explaining social isolation.

Predictor Variable	*β* Value	% of model runs retained
*Main Effects*		
Loneliness	**0.11**	**100**
Social Anhedonia	**0.21**	**100**
Social Avoidance		41.03
Depression		62.82
Nonsocial Cognition	**−0.06**	**80.77**
Social Cognition		55.12
*Group Interactions*		
Loneliness x Bipolar	**0.04**	**71.72**
Loneliness x Community	**0.18**	**97.43**
Social Anhedonia x Bipolar		55.13
Social Anhedonia x Community		39.74
Social Avoidance x Bipolar	**0.10**	**78.21**
Social Avoidance x Community		64.10
Depression x Bipolar		26.92
Depression x Community		44.87
Nonsocial Cognition x Bipolar	**0.05**	**70.51**
Nonsocial Cognition x Community		55.13
Social Cognition x Bipolar		60.25
Social Cognition x Community		46.15

LASSO regression model of schizophrenia, bipolar disorder, and community samples (R^2 ^= 0.33). Schizophrenia was used as the reference group in all interactions. An empty β value indicates no or practically no independent contribution of a predictor variable to social isolation above and beyond other variables. Presented on the right is the percentage of runs in which a predictor variable was retained in the model (i.e., its β value not shrunk to 0). The higher the percentage, the more robust the variable’s contribution.

*LASSO* Least Absolute Shrinkage and Selection Operator.

Variables in bold were retained in the final model.

For the across-group model of loneliness (Table 3), all variables showed non-zero main effects; higher scores on each variable were associated with greater loneliness. Once again, there were interactions between social isolation and loneliness in both BD and the CS. The model also showed an interaction between loneliness and depression in the CS, relative to schizophrenia.

**Table 3:** Explaining loneliness.

Predictor Variable	*β* value	% of model runs retained
*Main Effects*		
Social Isolation	**0.06**	**97.26**
Social Anhedonia	**0.29**	**100**
Social Avoidance	**0.14**	**90.41**
Depression	**0.12**	**93.15**
Nonsocial Cognition	**0.02**	**72.60**
Social Cognition	**0.05**	**97.26**
*Group Interactions*		
Social Isolation x Bipolar	**0.18**	**80.00**
Social Isolation x Community	**0.27**	**97.33**
Social Anhedonia x Bipolar		60.27
Social Anhedonia x Community		39.72
Social Avoidance x Bipolar		65.75
Social Avoidance x Community		65.75
Depression x Bipolar		60.27
Depression x Community	**0.12**	**91.78**
Nonsocial Cognition x Bipolar		42.47
Nonsocial Cognition x Community		43.83
Social Cognition x Bipolar		10.96
Social Cognition x Community		17.81

LASSO regression model of schizophrenia, bipolar disorder, and community samples (R^2^ = 0.47). Schizophrenia was used as the reference group in all interactions. An empty β value indicates no or practically no independent contribution of a predictor variable to loneliness above and beyond other variables. Presented on the right is the percentage of runs in which a predictor variable was retained in the model (i.e., its β value not shrunk to 0). The higher the percentage, the more robust the variable’s contribution.

*LASSO* Least Absolute Shrinkage and Selection Operator.

Variables in bold were retained in the final model.

### Follow-up analyses

As the R^2^ values for social isolation and loneliness in the schizophrenia sample were substantially lower than the values observed in BD and CS, we conducted follow-up LASSO regression analyses including additional variables which could be relevant for explaining social isolation and loneliness, particularly within schizophrenia. In these models, we included the original set of variables, as well as positive symptoms from the BPRS, negative symptoms from the CAINS (expressive and motivational subscales), mania symptom from the YMRS, and demographic variables (age and gender). Adding these variables in schizophrenia increased the R^2^ of social isolation to 0.39. The R^2^ for loneliness increased to 0.26. The most notable finding was the large influence of motivational negative symptoms, which explained the greatest proportion of variance in social isolation in all three samples. However, this finding was not surprising, as there is a high degree of conceptual and measurement overlap between the social isolation and motivational negative symptoms measures. The full model results for all three samples are in the Supplemental Material.

## Discussion

We used machine learning to identify predictors of isolation and loneliness in samples of schizophrenia, BD, and community members enriched for social isolation. Three main findings emerged. First, social anhedonia explained unique variance in social isolation and loneliness across all samples. Second, social isolation and loneliness were linked to each other in the BD and CS but not in schizophrenia. Third, nonsocial cognition explained unique variance in social isolation specifically in schizophrenia. Overall, these results offer possible targets for transdiagnostic and schizophrenia-specific interventions.

These results highlight the central and transdiagnostic role of social anhedonia. Greater social anhedonia was linked to social isolation and loneliness within and across schizophrenia, BD, and a community sample enriched for social isolation. While social anhedonia is linked to poor social functioning in schizophrenia^10–12,39,40^, this is the first study to our knowledge to show that it explains unique variance in social isolation and loneliness beyond social cognition, nonsocial cognition, depression, and social avoidance motivation. Similar patterns emerged in BD and CS, suggesting that these relationships are not unique to schizophrenia but exist across diagnoses.

Social isolation and loneliness were significantly more strongly associated in BD and CS than in schizophrenia. The relatively low association between the two variables in schizophrenia is closer to what is seen in unselected population samples, in which the associations are around 0.25^27,41^. Hence, the associations in BD and CS were unusually high. There are a few possible reasons for this. Emotional reactions to social isolation may be more salient and cognitively accessible in patients with BD and possibly CS. These groups may be more aware of prior experiences involving social isolation and associated emotional reactions (e.g., loneliness) than those with schizophrenia^13,42^. In BD specifically, social avoidance played a role in both isolation and loneliness, something not observed in the other samples. Social avoidance in BD may be a byproduct of high sensitivity to social rejection, which could contribute to greater social isolation and loneliness^42^.

Nonsocial cognition explained unique variance in social isolation in the schizophrenia sample but not in BD or CS. Deficits in nonsocial cognition are core components of schizophrenia and typically precede the onset of psychosis by several years^43^—a feature that is not reliably found with BD^44,45^. Hence, it is not surprising that nonsocial cognition was linked to social isolation in schizophrenia, but its lack of association with loneliness was notable. One study has reported a link between nonsocial cognition and loneliness, but it did not account for the other variables modeled in the current study^46^. Several variables were associated with loneliness in schizophrenia, suggesting that nonsocial cognition does not add explanatory power once accounting for other relevant variables, as it does for isolation. Another notable finding was the presence of an interaction between loneliness and depression in the CS, relative to schizophrenia. This result suggests that loneliness could be more strongly connected to disruptions in mood in non-clinical samples than in schizophrenia, where other clinical and contextual factors may influence the association. Lastly, in the follow-up analyses, we found that motivational negative symptoms from the CAINS had the strongest link to social isolation in all three samples. This finding is not surprising and may even be expected given that a main component of the motivational negative symptoms subscale centers around the feelings and overt behavior regarding social contact. In other words, having low motivation for social contact is associated with a higher degree of social isolation.

Our main findings offer suggestions for both transdiagnostic and illness-specific interventions. Social anhedonia is a clear target for potential interventions aimed at improving social isolation and loneliness. It was the only variable to predict both forms of social disconnection across samples, suggesting that interventions for social anhedonia might help reduce social isolation and loneliness regardless of diagnosis. One existing relevant intervention is combined motivational interviewing and cognitive-behavioral therapy. This approach has recently been shown to improve motivational negative symptoms in schizophrenia and may have additional clinical utility in decreasing social isolation and loneliness^47^. Further, schizophrenia may benefit from a combined treatment focused on social anhedonia and nonsocial cognition. Lastly, social avoidance may have specific utility as a treatment target for social isolation in bipolar disorder. Virtual reality-based interventions focused on reducing loneliness and social anxiety could be a promising future direction^48^.

The study had several limitations. A major limitation is that the data were cross-sectional and thus prevented us from drawing any causal conclusions about the results. Hence, even though we were able to show that specific variables explain significant variability in social isolation and loneliness, it remains to be seen whether they are the antecedents or consequences of social disconnection. Moreover, although the comparability of social isolation across groups is a strength of the study, the CS is not representative of the community at large due to the selection method. Specifically, the CS was only enriched for social isolation and not loneliness, making the extent to which these results translate to samples with high levels of loneliness unclear. Another limitation is that the social anhedonia and social avoidance measures were based on self-report. Observational or performance-based measures, such as social effort-based decision-making tasks^49^, could provide more sensitive indicators. Relatedly, we used a composite measure of the three social cognitive tasks instead of examining their unique effects. We chose to use a composite instead of individual tasks because of our modest sample sizes, along with the fact that some of the tasks (e.g., facial affect identification) are not designed for high reliability at the individual trial level. We also did not assess other domains that could potentially impact social isolation and loneliness, such as attribution biases. Additionally, the BD sample size was relatively small. Although LASSO can help overcome limitations of unequal sample sizes among the groups through regularization and cross-validation, the usual limitations of small samples still apply (e.g., generalization and power). Lastly, we accounted for less variance in social isolation and loneliness in schizophrenia compared to the amount accounted for in BD and CS. Thus, there is a need for larger machine learning studies that can include other potentially relevant variables that may help explain social disconnection in schizophrenia. One promising future direction in this area is to use social network analysis, which could help identify specific aspects of social networks (e.g., network interconnectedness) that contribute to social isolation and loneliness^50^.

Social isolation and loneliness are associated with substantial public health risks. The present study used machine learning to identify social anhedonia as a transdiagnostic variable that is linked to social isolation and loneliness in schizophrenia, BD, and a CS enriched for social isolation. Pending replication in larger studies, social anhedonia may prove to be a particularly important target for the development or adaptation of interventions to address isolation and loneliness across multiple psychiatric groups and the broader community.

## Supplementary information

Supplemental Material

## Data Availability

Data used in this study is available upon request.
