# Supplementary material for: Using machine learning to understand social isolation and loneliness in schizophrenia, bipolar disorder, and the community
Source: Schizophrenia (Heidelb). 2024 Oct 5;10(1):88. doi: 10.1038/s41537-024-00511-y (PMC11455897; doi:10.1038/s41537-024-00511-y)
Supplement: Supplementary file 1 — Supplemental Material [file 41537_2024_511_MOESM1_ESM.docx]

**Supplemental Material for Using machine learning to understand social isolation and loneliness in schizophrenia, bipolar disorder, and the community**

Samuel J. Abplanalp, PhD^*1,2,3^

Michael F. Green, PhD^3,2,1^

Jonathan K. Wynn, PhD^1,2,3^

Naomi I. Eisenberger, PhD^4^

William P. Horan, PhD^3,5^

Junghee Lee, PhD^6^

Amanda McCleery, PhD^3,7^

David J. Miklowitz, PhD^3^

L. Felice Reddy, PhD^2,3,8^

Eric A. Reavis, PhD^3,2,1^

1. VA Greater Los Angeles Healthcare System, Los Angeles, CA, USA
2. VA Rehabilitation R&D Center on Enhancing Community Integration for Homeless Veterans, Los Angeles, CA, USA
3. Department of Psychiatry and Biobehavioral Sciences, Jane and Terry Semel Institute for Neuroscience and Human Behavior, UCLA, Los Angeles, CA, USA
4. Department of Psychology, UCLA, Los Angeles, CA, USA
5. Karuna Therapeutics, Boston, MA, USA
6. Department of Psychiatry and Behavioral Neurobiology, University of Alabama at Birmingham, Birmingham, AL, USA
7. Department of Psychological and Brain Sciences, University of Iowa, Iowa City, IA, USA
8. Department of Psychiatry, University of North Carolina, Chapel Hill, NC, USA

^*^To whom correspondence should be addressed: Samuel J. Abplanalp, VA Greater Los Angeles Healthcare System, MIRECC 210A, Bldg. 206, 11301 Wilshire Blvd, Los Angeles, CA 90073, United States; Phone: 310-478-3711; 44041; Email: sabplanalp@mednet.ucla.edu

Supplemental Table 1: Explaining social isolation. Within-group LASSO regression models of schizophrenia, bipolar disorder, and the community sample.

Supplemental Table 2: Explaining loneliness. Within-group LASSO regression models of schizophrenia, bipolar disorder, and the community sample.

| Supplemental Table 1. Explaining social isolation. Within-group LASSO regression models of schizophrenia, bipolar disorder, and the community sample. | | | | | | |
| --- | --- | --- | --- | --- | --- | --- |
| Predictor Variable | β Value | | | | % of model runs retained |  |
| *Schizophrenia (R^2^ = 0.39)* |  | | | |  |  |
| Loneliness |  | | | | 75.75 |  |
| **Social Anhedonia** | **0.13** | | | | **90.91** |  |
| Social Avoidance |  | | | | 71.21 |  |
| Depression |  | | | | 39.39 |  |
| Nonsocial Cognition |  | | | | 22.72 |  |
| Social Cognition |  | | | | 60.61 |  |
| **Motivational Negative Symptoms** | | **0.40** | | **100** | |  |
| Expressive Negative Symptoms |  | | | | 72.72 |  |
| Positive Symptoms |  | | | | 22.72 |  |
| **Mania Symptoms** | **0.10** | | | | **87.88** |  |
| Gender |  | | | | 71.21 |  |
| Age |  | | | | 0.00 |  |
| Predictor Variable | β Value | | | | % of model runs retained |  |
| *Bipolar disorder (R^2^ = 0.50)* | | | | | |  |
| **Loneliness** | **0.12** | | | | **100** |  |
| **Social Anhedonia** | **0.01** | | | | **61.43** |  |
| **Social Avoidance** | **0.12** | | | | **94.28** |  |
| **Depression** | **0.04** | | | | **88.57** |  |
| Nonsocial Cognition |  | | | | 68.57 |  |
| Social Cognition |  | | | | 45.71 |  |
| **Motivational Negative Symptoms** | | **0.18** | | | **100** |  |
| Expressive Negative Symptoms |  | | | | 65.71 |  |
| Positive Symptoms |  | | | | 64.28 |  |
| **Mania Symptoms** | **0.03** | | | | **84.29** |  |
| Gender |  | | | | 65.57 |  |
| Age |  | | | | 48.57 |  |
| Predictor Variable | β Value | | | | % of model runs retained |  |
| *Community sample (R^2^ = 0.54)* |  | | | |  |  |
| **Loneliness** | **0.16** | | | | **93.93** |  |
| **Social Anhedonia** | **0.11** | | | | **90.90** |  |
| Social Avoidance |  | | | | 45.45 |  |
| Depression |  | | | | 62.12 |  |
| Nonsocial Cognition |  | | | | 50.00 |  |
| Social Cognition |  | | | | 65.15 |  |
| **Motivational Negative Symptoms** | | | **0.36** | | **100** |  |
| Expressive Negative Symptoms |  | | | | 65.15 |  |
| Positive Symptoms |  | | | | 39.39 |  |
| Mania Symptoms |  | | | | 59.10 |  |
| Gender |  | | | | 63.33 |  |
| Age |  | | | | 66.67 |  |
| *Note*. LASSO = Least Absolute Shrinkage and Selection Operator. An empty β value indicates no or practically no independent contribution of a predictor variable to loneliness above and beyond other variables. Presented on the right is the percentage of runs in which a predictor variable was retained in the model (i.e., its β value not shrunk to 0). The higher the percentage, the more robust the variable’s contribution. | | | | | |  |
| Supplemental Table 2. Explaining loneliness. Within-group LASSO regression models of schizophrenia, bipolar disorder, and the community sample. | | | | | | |
| Predictor Variable | β Value | | | | % of model runs retained |  |
| *Schizophrenia (R^2^ = 0.26)* |  | | | |  |  |
| Social Isolation |  | | | | 74.24 |  |
| **Social Anhedonia** | **0.25** | | | | **100** |  |
| **Social Avoidance** | **0.03** | | | | **84.33** |  |
| **Depression** | **0.07** | | | | **89.39** |  |
| Nonsocial Cognition |  | | | | 74.24 |  |
| **Social Cognition** | **0.03** | | | | **84.29** |  |
| Motivational Negative Symptoms | |  | | 51.50 | |  |
| Expressive Negative Symptoms |  | | | | 53.03 |  |
| **Positive Symptoms** | **0.03** | | | | **84.88** |  |
| Mania Symptoms |  | | | | 69.67 |  |
| **Gender** | **-0.11** | | | | **84.84** |  |
| Age |  | | | | 75.75 |  |
| Predictor Variable | β Value | | | | % of model runs retained |  |
| *Bipolar disorder (R^2^ = 0.53)* | | | | | |  |
| **Social Isolation** | **0.26** | | | | **100** |  |
| **Social Anhedonia** | **0.08** | | | | **86.36** |  |
| **Social Avoidance** | **0.19** | | | | **87.88** |  |
| **Depression** | **0.16** | | | | **96.97** |  |
| Nonsocial Cognition |  | | | | 46.96 |  |
| Social Cognition |  | | | | 45.12 |  |
| **Motivational Negative Symptoms** | | **0.16** | | | **92.42** |  |
| **Expressive Negative Symptoms** | **-0.10** | | | | **80.30** |  |
| Positive Symptoms |  | | | | 63.67 |  |
| Mania Symptoms |  | | | | 62.12 |  |
| Gender |  | | | | 66.67 |  |
| **Age** | **-0.19** | | | | **87.57** |  |
| Predictor Variable | β Value | | | | % of model runs retained |  |
| *Community sample (R^2^ = 0.59)* |  | | | |  |  |
| **Social Isolation** | **0.31** | | | | **100** |  |
| **Social Anhedonia** | **0.27** | | | | **100** |  |
| **Social Avoidance** | **0.17** | | | | **90.90** |  |
| **Depression** | **0.22** | | | | **93.93** |  |
| Nonsocial Cognition |  | | | | 24.22 |  |
| **Social Cognition** | **0.01** | | | | **65.15** |  |
| Motivational Negative Symptoms | | |  | | 40.90 |  |
| Expressive Negative Symptoms |  | | | | 42.22 |  |
| Positive Symptoms |  | | | | 62.12 |  |
| Mania Symptoms |  | | | | 24.22 |  |
| Gender |  | | | | 51.55 |  |
| Age |  | | | | 46.95 |  |
| *Note*. LASSO = Least Absolute Shrinkage and Selection Operator. An empty β value indicates no or practically no independent contribution of a predictor variable to loneliness above and beyond other variables. Presented on the right is the percentage of runs in which a predictor variable was retained in the model (i.e., its β value not shrunk to 0). The higher the percentage, the more robust the variable’s contribution. | | | | | |  |
